# The fine tuning of metabolism, autophagy and differentiation during *in vitro* myogenesis

**DOI:** 10.1038/cddis.2016.50

**Published:** 2016-03-31

**Authors:** P Fortini, C Ferretti, E Iorio, M Cagnin, L Garribba, D Pietraforte, M Falchi, B Pascucci, S Baccarini, F Morani, S Phadngam, G De Luca, C Isidoro, E Dogliotti

**Affiliations:** 1 Department of Environment and Primary Prevention, Molecular Epidemiology Unit, Istituto Superiore di Sanità, Rome, Italy; 2 Department of Cell Biology and Neurosciences, Istituto Superiore di Sanità, Rome, Italy; 3 Department of Health Sciences, Università del Piemonte Orientale 'Amedeo Avogadro', Novara, Italy; 4 National AIDS Center, Istituto Superiore di Sanità, Rome, Italy; 5 Institute of Crystallography, Consiglio Nazionale delle Ricerche, Rome, Italy; 6 Department of Haematology, Oncology and Molecular Medicine, Istituto Superiore di Sanità, Rome, Italy

## Abstract

Although the mechanisms controlling skeletal muscle homeostasis have been identified, there is a lack of knowledge of the integrated dynamic processes occurring during myogenesis and their regulation. Here, metabolism, autophagy and differentiation were concomitantly analyzed in mouse muscle satellite cell (MSC)-derived myoblasts and their cross-talk addressed by drug and genetic manipulation. We show that increased mitochondrial biogenesis and activation of mammalian target of rapamycin complex 1 inactivation-independent basal autophagy characterize the conversion of myoblasts into myotubes. Notably, inhibition of autophagic flux halts cell fusion in the latest stages of differentiation and, conversely, when the fusion step of myocytes is impaired the biogenesis of autophagosomes is also impaired. By using myoblasts derived from p53 null mice, we show that in the absence of p53 glycolysis prevails and mitochondrial biogenesis is strongly impaired. P53 null myoblasts show defective terminal differentiation and attenuated basal autophagy when switched into differentiating culture conditions. In conclusion, we demonstrate that basal autophagy contributes to a correct execution of myogenesis and that physiological p53 activity is required for muscle homeostasis by regulating metabolism and by affecting autophagy and differentiation.

Muscle satellite cells (MSCs) in adult muscle remain quiescent until external stimuli (such as injury or even exercise) trigger their re-entry into the cell cycle. Their progeny, myoblasts, fuse to form new multinucleated myofibers. In this study, the ability of MSC to give rise to muscle progenitor cells (that is, myoblasts) that could differentiate and fuse *in vitro* has been exploited to analyze the integrated network of signaling pathways that operate during myogenesis.

Autophagy undergoes a fine tuning during cell and tissue differentiation in order to adapt to the dynamic changes occurring in the tissue microenvironment.^1^ Using the stable C2C12 cell line, it was shown that autophagy is induced during muscle differentiation despite the concomitant activation of mammalian target of rapamycin (mTOR).^2^ Interestingly, inhibition of autophagy was found to impair the differentiation and fusion of C2C12 myoblasts, while favoring their apoptosis.^3^ Autophagy is increased in muscle in several physiological and pathological conditions, including fasting, atrophy and exercise.^4^ Much less is known about the link between cell metabolism and autophagy during muscle differentiation under physiological conditions.

p53 has been shown to promote myoblast differentiation by regulating the function of pRb,^5, 6^ and to have a pleiotropic role in muscle metabolism by promoting exercise-induced mitochondrial biogenesis in skeletal muscle.^7, 8^ How the physiological level of p53 impacts on these changes during differentiation has not been explored.

The role of p53 in the regulation of autophagy is multi-facets.^9^ Nuclear p53 positively regulates autophagy following exogenous stress, resulting in a pro-death or pro-survival outcomes.^10^ Conversely, cytoplasmic p53 inhibits autophagy under starvation or endoplasmic reticulum (ER) stress.^11^ What is the role of p53 in the regulation of basal autophagy during myogenesis and its physiological implications are still unknown.

We address these issues using mouse skeletal MSC-derived myoblasts that when differentiate *in vitro* well mimic the dynamic processes occurring *in vivo* when a myoblast is asked to differentiate and fuse into a fully differentiated myotube. The findings here reported unravel a clear role for basal autophagy in muscle differentiation and identify a role for p53 in muscle metabolism and basal autophagy.

## Results

### Metabolic remodeling and mitochondrial biogenesis occur during differentiation

**Figure 1:**
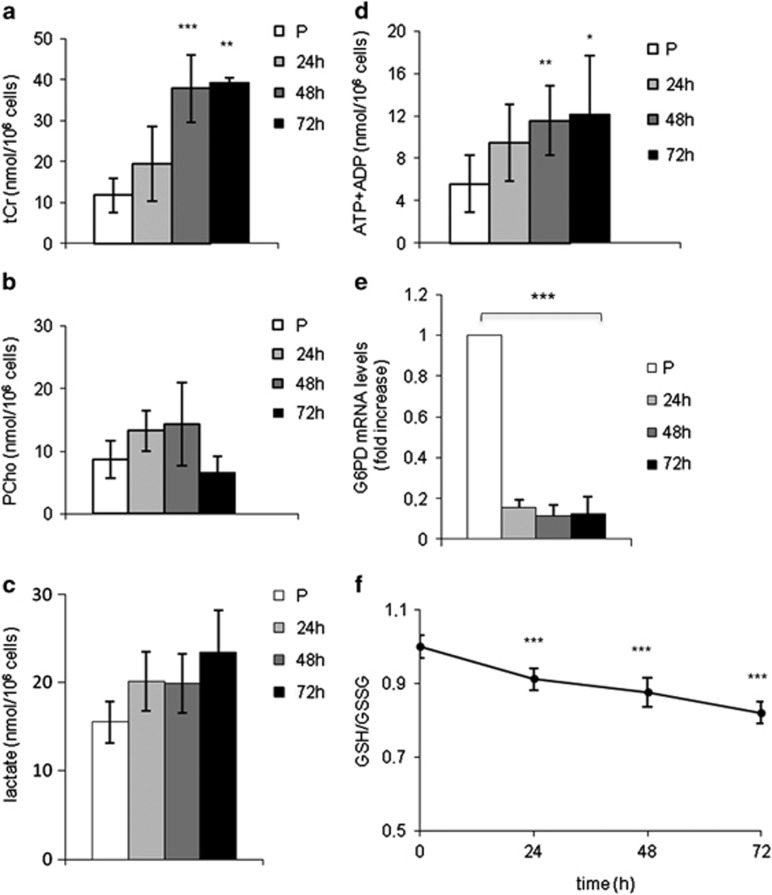
The metabolic profile changes during myogenesis. The concentration of acqueous metabolites, as measured by 1H-NMR, is reported as nmol/10^6^ cells (mean ±S.D. for *n*⩾3) for (**a**) tCr (creatine plus phosphocreatine), (**b**) phosphocholine (PCho), (**c**) lactate and (**d**) intracellular level of ATP+ ADP. (**e**) mRNA levels of G6PD during differentiation as determined by qRT-PCR. Expression levels were normalized *versus* two housekeeping genes, HGPRT and 18 S. Relative expression levels are shown with respect to the basal level of the proliferating myoblasts, which is set equal to 1 (mean ±S.D. for *n*⩾3). (**f**) GSH and GSSG ratio obtained by enzymatic assay titration (mean ±S.D. for *n*≥3). Statistical analysis was performed to compare the values obtained in proliferating myoblasts (P) with those obtained in differentiated cells (24–72 h in DM). *P-value <0.05; **P<0.01; ***P<0.001

The metabolic profile during myogenesis was analyzed by 1H-NMR in mouse MSC-derived myoblasts induced to differentiate *in vitro*. The relevant changes in the levels of specific metabolites are presented in Figure 1 and the complete spectrum of metabolites is shown in Supplementary Table S1A. Remarkably, the activation of muscle creatine kinase and the decreased requirement for phospholipid synthesis that occur during myogenesis *in vivo* were faithfully reproduced *in vitro*, as shown by the increased levels of total creatine (tCr) (Figure 1a) and decreased levels of phosphocholine (PCho) (Figure 1b) at the latest differentiation time. During differentiation, the absolute concentration of lactate did not significantly change (Figure 1c). A slight increase of the absolute concentration of ATP+ADP (Figure 1d) was observed whereas AMP levels were relatively stable. (Supplementary Table S1A).

**Figure 2:**
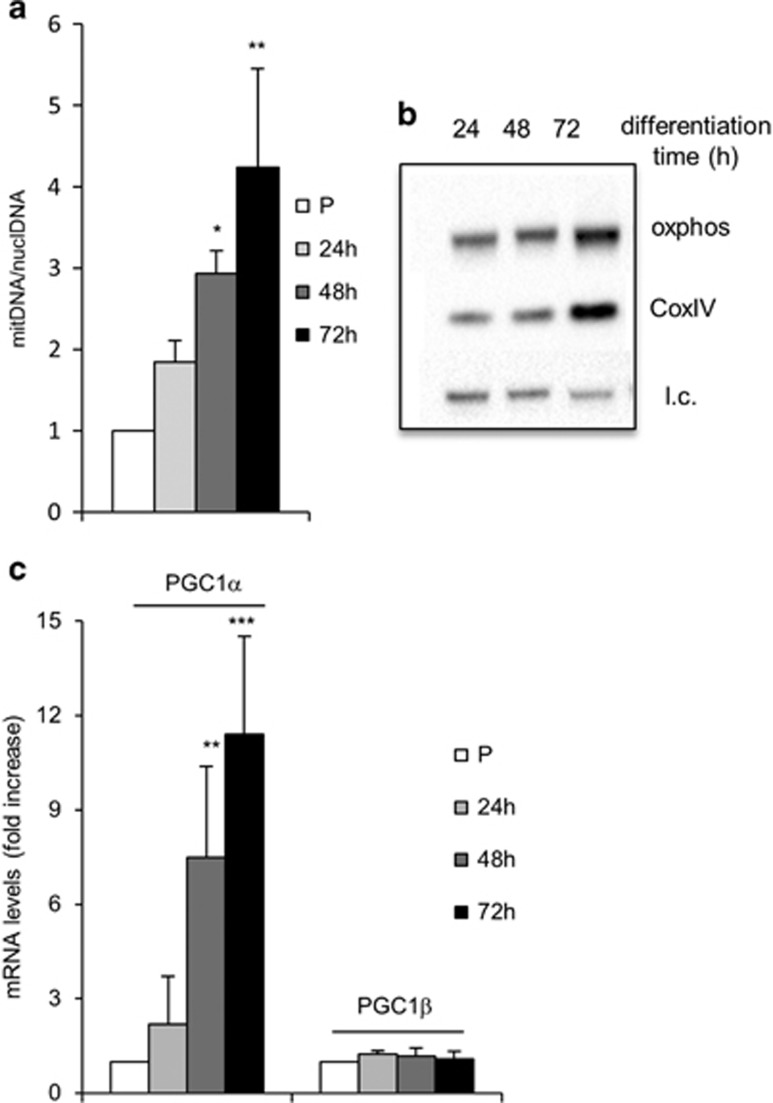
Mitochondrial biogenesis is upregulated during differentiation. (**a**) Mitochondrial *versus* nuclear DNA was determined by absolute quantitative RT-PCR. (**b**) WB analysis was performed with anti-oxphos and anti-CoxIV antibodies on whole-protein extracts; 20 *μ*g of total protein were loaded in each lane; l.c.=loading control. (**c**) Levels of mRNA transcripts of PGC1s were determined by comparative qRT-PCR. Expression levels were normalized *versus* two housekeeping genes, HGPRT and 18 S. In (**c**) and (**e**). the reported values are the means of three independent experiments±S.D.; the value of proliferating myoblasts (P) is set equal to 1. *P-value <0.05; **P<0.01; ***P<0.001

The pentose phosphate pathway (PPP) is not very active in muscle cells and is upregulated in pathophysiological conditions.^12^ Indeed, the mRNA levels of glucose-6-phosphate dehydrogenase (G6PD) decreased during differentiation (Figure 1e) and the ratio between reduced (GSH) and oxidized (GSSG) glutathione also decreased (Figure 1f). The increase in the level of glutathione as measured by NMR in myoblasts *versus* myotubes (Supplementary Table S1A) was confirmed by the significant increase in the levels of both GSH and GSSG during the myoblast–myotube transition (data not shown). Mitochondrial (mt) DNA molecules (Figure 2a) and the levels of mitochondrial proteins, such as the cytochrome c oxidase (COXIV) and the succinate ubiquinone oxydoreductase (oxphos) (Figure 2b) significantly increased during myogenesis. Peroxisome proliferator-activated receptor gamma coactivator 1 alpha (PGC-1*α*) is significantly upregulated in the course of muscle differentiation, whereas peroxisome proliferator-activated receptor gamma coactivator 1 beta (PGC-1*β*) remained unchanged (Figure 2c).

In conclusion, the metabolic profile of our *in vitro* cell system well mimics what occurs in the skeletal muscle *in vivo* showing that during differentiation aerobic metabolism greatly increases, whereas the PPP is downregulated.

### Autophagy is activated during myogenesis

When proliferating myoblasts were switched to grow in differentiation medium (DM), the transcript levels of autophagy-related genes, such as light chain 3 (LC3) and forkhead box O3 (FOXO3), increased (Supplementary Figures S1A and B) suggesting that autophagy may take place during differentiation.

**Figure 3:**
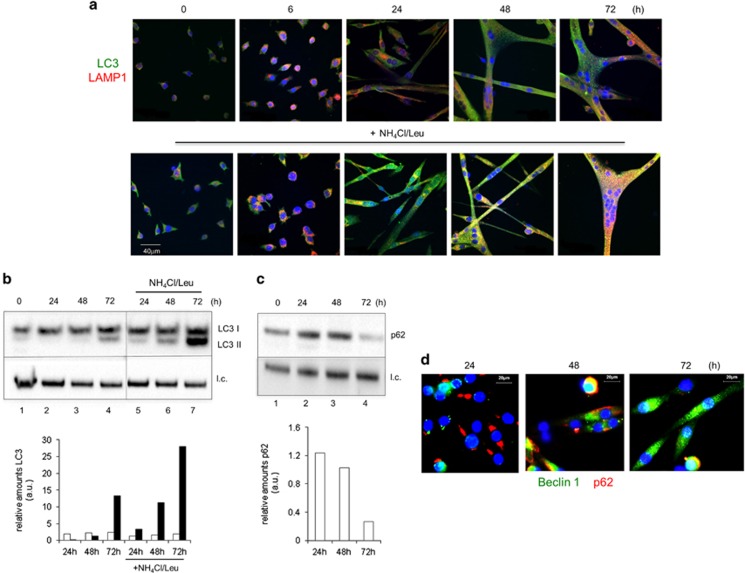
Autophagy is induced during skeletal muscle differentiation. (**a**) Representative fluorescence confocal microscopy images of wild-type muscle cells during differentiation: LC3 (green) and Lamp1 (red). Myoblasts were grown in DM and fixed at different differentiation times (0, 6, 24, 48 and 72 h). (**b**) Top: WB analysis of the autophagy markers LC3-I (18 kDa) and LC3-II (16 kDa), and (**c**) p62 (62 kDa) in wild-type muscle cells grown in DM for different periods of time (0, 24, 48 and 72 h). The autophagic flux inhibitors, ammonium chloride and leupeptin (NH_4_Cl/Leu, 20 mM/100 *μ*M), were added 2 h before cell harvest; l.c.=loading control. (**b** and **c**) Bottom: fold changes of the indicated proteins relative to the l.c. were determined by densitometric scanning. The values are normalized for the levels in proliferating cells. One experiment out of at least three independent experiments is shown. (**d**) Representative fluorescence confocal microscopy images of wild-type muscle cells during differentiation (24, 48 and 72 h in DM): Beclin 1 (green) and p62 (red). Nuclei are stained with DAPI

Autophagy was investigated by immunofluorescence (IF) and western blotting (WB) analyses in differentiating myoblasts. IF staining of LC3, a hallmark of autophagosomes,^13^ and of the lysosome-associated membrane protein, Lamp1, indicated that autophagy is active since the first 24 h of culture in DM (Figure 3a, top). When lysosomal inhibitors were used, the accumulation of LC3-positive vacuoles was observed (Figure 3a, bottom). The colocalization of LC3 and Lamp1 and the increased LC3 fluorescence in the presence of inhibitors confirm the induction of autophagy. Figure 3b shows WB and densitometric analysis of LC3-I to LC3-II conversion that negligible in myoblasts (lane 1), whereas it increases during differentiation time (lanes 3–4). Under exposure to lysosomal inhibitors (lanes 5–8), the progressive accumulation of the LC3-II isoform upon switching the culture into the DM confirmed the occurrence of autophagic flux during myogenesis. WB (Figure 3c) and IF analysis (Figure 3d) of p62 reflects lysosomal proteolysis during muscle differentiation indicative of ongoing autophagy flux. Autophagic flux was confirmed by using myogenic precursor cells freshly isolated (1-month-old mice) (Supplementary Figure S2).

### Autophagy is required to accomplish myotube fusion

**Figure 4:**
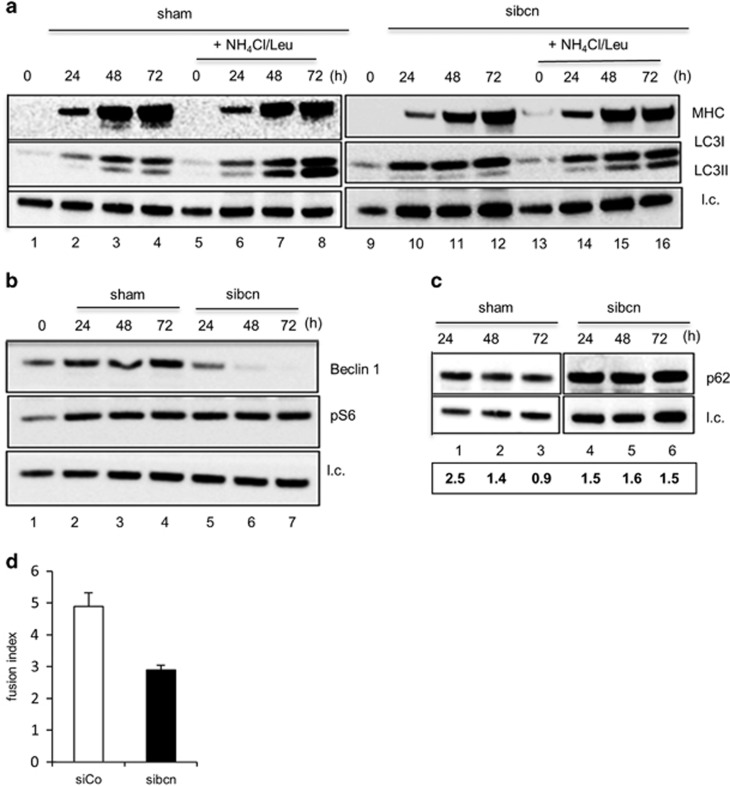
Downregulation of Beclin 1 inhibits differentiation-associated autophagy and affects the fusion step. WB analysis of MHC (200 kDa) and LC3-I (18 kDa) and II (16 kDa) (**a**), pS6 (32 kDa) and Beclin 1 (60 kDa) (**b**) and p62 (62 kDa) (**c**) in whole-cell extracts from muscle cells either untreated (sham, Interferin only) or treated with 20 mM Beclin 1 small interfering RNAs (siBecn). The interference procedure was carried out in proliferating myoblasts that immediately after transfection were shifted in DM for different periods of time (0, 24, 48 and 72 h). A mix of ammonium chloride and leupeptin (NH_4_Cl/Leu, 20 mM/100 *μ*M) was added to the medium 2 h before cell harvest as indicated; l.c.=loading control. In panel (**c**) (bottom), the values of the densitometric scanning of the bands of p62 *versus* l.c. are provided. (**d**) Fusion index of sham (Interferin only) and Beclin 1-silenced terminally differentiated (TD) muscle cells (mean ±S.D.)

**Figure 5:**
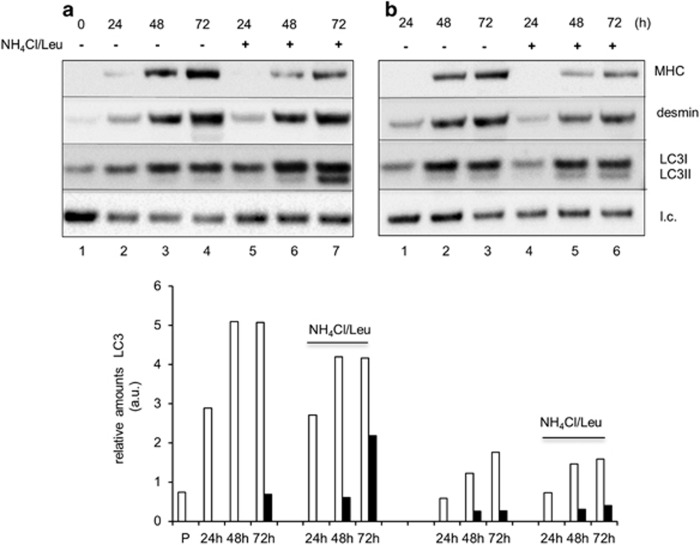
Autophagy is required for the fusion of myocytes into multinucleated myotubes. WB analysis of MHC (200 kDa), desmin (53 kDa) and LC3-I (18 kDa) and II (16 kDa) in whole-cell extracts from muscle cells allowed to terminally differentiate and fuse in multinucleated myotubes (**a**) or to reach the status of fully differentiated myocytes by preventing cell contact (**b**). A mix of ammonium chloride and leupeptin (NH_4_Cl/Leu, 20 mM/100 *μ*M) was added to the medium 2 h before cell harvest as indicated. (**a** and **b**) Bottom: fold changes of the indicated proteins relative to the loading control (l.c.) were determined by densitometric scanning. One representative experiment (out of two) is shown

To investigate the functional cross-talk between autophagy and completion of muscle differentiation, we adopted three strategies. First, we attempted to inhibit autophagy genetically: Beclin 1 (Becn1), a key activator of Vps34 (ref. 14) was silenced by targeted siRNA (Figure 4). Complete knockdown of Becn1 expression, *versus* sham-counterpart, was achieved only between 48 and 72-h differentiation time (Figure 4b, lanes 6–7). Progressive LC3-II accumulation occurring in sham-transfected cultures (Figure 4a, lanes 5–8) in the presence of lysosomal inhibitors was significantly reduced in siBecn1 cultures (lanes 9–16) and, consistently, p62 degradation was impaired when Becn1 was silenced (Figure 4c, lanes 4–6). Phosphorylation of the ribosomal S6 subunit (pS6) is observed during myogenesis of both normal and siBecn cultures (Figure 4b), suggesting that differentiation-associated autophagy is mammalian target of rapamycin complex 1 (mTORC1) inactivation independent (see below). Remarkably, the fusion index between 48 and 72 h in siBecn-transfected cultures was reduced by 1.7-fold (Figure 4d), whereas the differentiation index was unaffected (data not shown). Disappointingly, siRNA targeting of ATG5 was unable to inhibit autophagy, likely due to residual (approximately 20% expression) but sufficient levels of protein to trigger autophagy.^15^ To corroborate these findings, a straightforward strategy was used. If the fusion step is associated with the induction of autophagy, it is expected that the autophagic flux is reduced in myocytes that are fully differentiated but not fused into syncytial myotubes. Although in myotubes (Figure 5a), LC3-II accumulated in the presence of inhibitors (lanes 5–7), consistent with ongoing autophagic flux, this accumulation was not observed in myocytes (Figure 5b, lanes 4–6), thus indicating a block in the biogenesis of autophagosomes in the absence of fusion.

**Figure 6:**
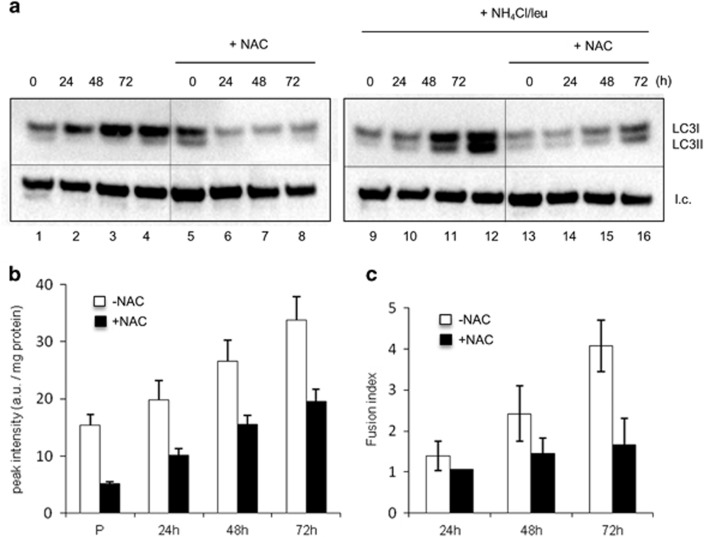
Inhibition of autophagy by NAC affects the fusion step. (**a**) WB analysis of LC3-I (18 kDa) and II (16 kDa) in whole-cell extracts from muscle cells either untreated (left panel) or exposed to a mix of ammonium chloride and leupeptin (NH_4_Cl/Leu, 20 mM/100 *μ*M) added to the medium 2 h before cell harvest (right panel); l.c.=loading control. (**b**) ROS levels as detected by EPR (relative intensity of CP^•^) in muscle cells either proliferating (P) or during differentiation (24, 48 and 72 h in DM) with and without NAC addition (5 mM f.c.) to the medium. (**c**) Fusion index of muscle cells at different differentiation times (24, 48 and 72 h in DM) with and without NAC addition (5 mM f.c.) to the medium (mean ±S.D.; *n*=3). Without NAC: white boxes; with NAC: black boxes. This concentration of NAC (5 mM) inhibited by about 18% the intensity of 0.5 mM pre-formed stable CP^•^ after 20 min at 37 °C

Finally, as the general antioxidant *N*-acetyl cysteine (NAC) has been shown to significantly decrease the basal autophagic flux in skeletal muscles of mice,^16^ we explored whether the inhibition of autophagy by this means would also induce a block in differentiation (Figure 6). In all, 5 mM NAC effectively inhibited the basal autophagic flux (Figure 6a). Remarkably, under NAC incubation, the fusion index significantly decreased by approximately 2.5-fold (Figure 4c), providing further evidence that basal autophagy is strictly associated with a successful fusion step. It is of note that ROS levels, as measured by EPR, which showed a progressive increase during differentiation were significantly reduced by the addition of NAC (Figure 6b).

### Autophagy in differentiating muscle cells is independent of mTORC1 inactivation

**Figure 7:**
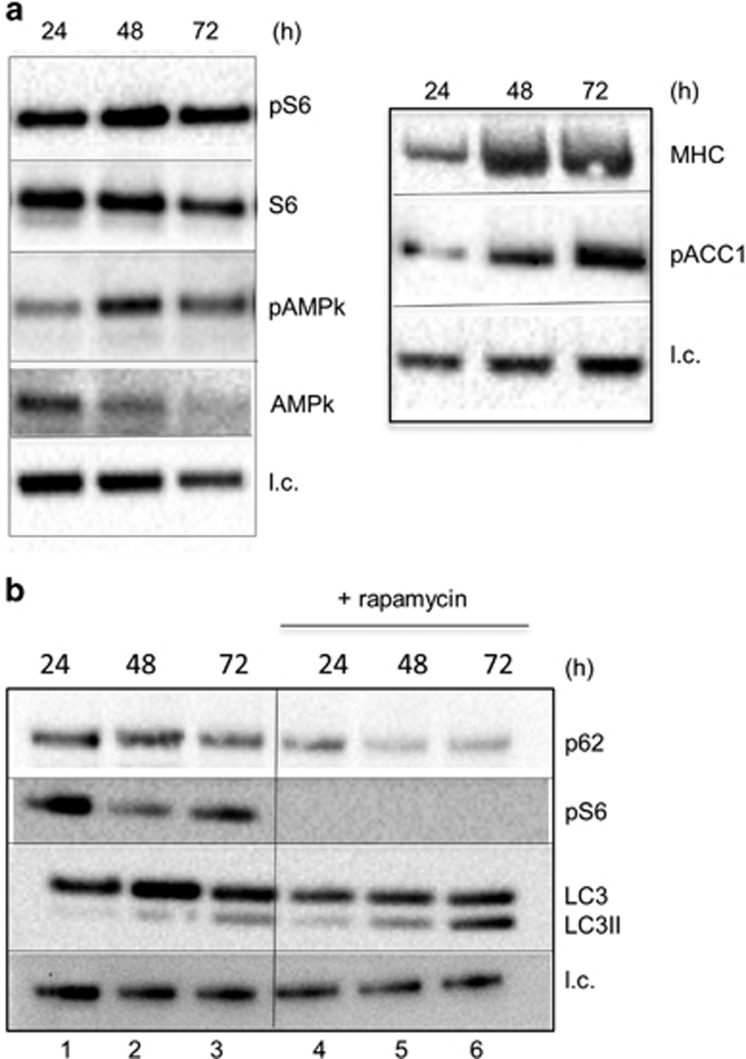
Basal autophagy is mTORC1 inactivation independent and is associated with AMPK activation during myogenesis. (**a**) WB analysis of pS6, S6 (32 kDa), pAMPk and AMPK (62 kDa) (left panel) and MHC (200 kDa) and p-ACC (280 kDa) (right panel) in whole-cell extracts during differentiation (24, 48 and 72 h in DM); l.c.=loading control. (**b**) WB analysis of MHC (200 kDa), p62 (62 kDa), desmin (53 kDa), pS6 (32 kDa), LC3-I (18 kDa) and LC3-II (16 kDa) in whole-cell extracts from muscle cells during differentiation in standard culturing conditions or in the presence of 5 mM rapamycin (rap)

By using pS6, as a surrogate of mTORC1 activity, it appears that this pathway is active during differentiation, although a progressive decrease of total S6 is observed during differentiation (Figure 7a). In the same time window, the levels of the Thr172 phosphorylated form of 5' adenosine monophosphate-activated protein kinase (pAMPk) (Figure 7a) and the AMPk downstream target acetyl CoA carboxylase (pACC1) (Figure 7b) increased during differentiation time.

To verify that basal autophagy was activated at the maximal level, we inhibited mTOR with rapamycin (Figure 7b). The inhibition of mTOR-dependent protein synthesis branch was testified by the absence of pS6 in differentiating cells (lanes 4–6). Rapamycin induced the accumulation of LC3-II and the concomitant degradation of p62 (lanes 4–6), indicating that autophagy was further stimulated when mTOR was inhibited. The combination of these two events indicates that under this condition, the autophagic flux was accelerated along with a great stimulation of autophagosome formation. In fact, degradation of p62 was faster in rapamycin-treated cells, concomitantly to a progressive LC3-II accumulation (compare lanes 1–3 with lanes 4–6). A marked inhibition of fusion was observed in the presence of rapamycin (data not shown), consistent with a previous report indicating that mTOR inactivation is detrimental for myogenesis via regulation of fusion factors.^17^

### Lack of p53 affects cell metabolism, basal autophagy and myogenesis

**Figure 8:**
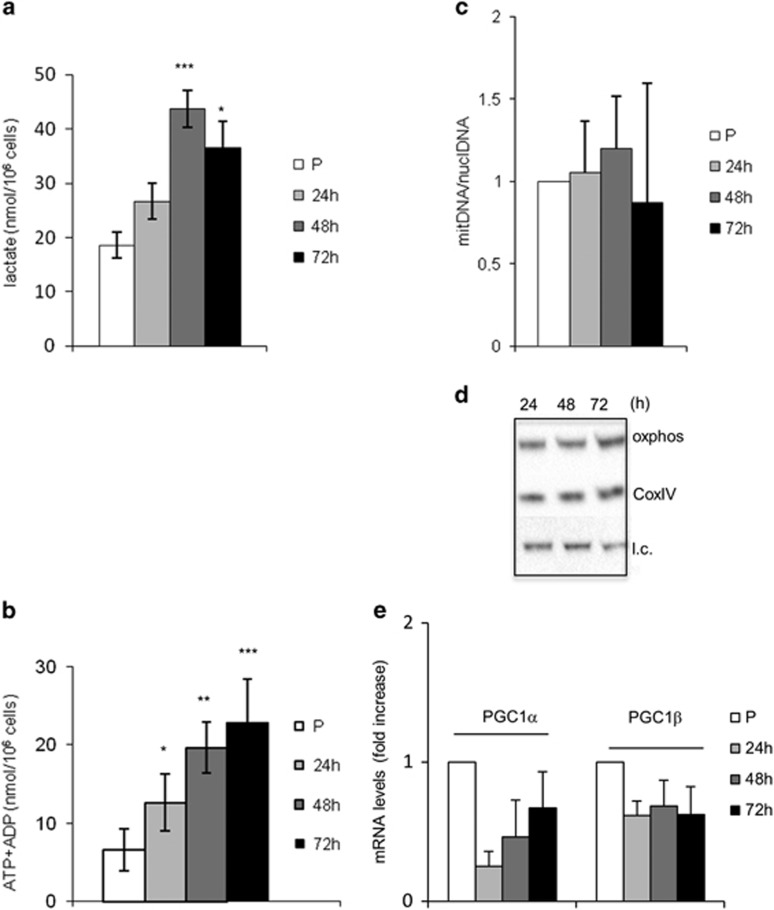
The metabolic profile and mitochondrial biogenesis are affected in the absence of p53. The concentration of acqueous metabolites, as measured by 1H-NMR in p53 null muscle cells, is reported as nmol/10^6^ cells (mean ±S.D. for *n*≥3) for (**a**) lactate, and (**b**) intracellular level of ATP+ ADP (8.53 p.p.m.). (**c**) Mitochondrial *versus* nuclear DNA was determined by absolute quantitative RT-PCR. (**d**) WB analysis was performed with anti-oxphos and anti-CoxIV antibodies on whole-protein extracts; 20 *μ*g of total protein were loaded in each lane; l.c.=loading control. (**e**) Levels of mRNA transcripts of PGC1s were determined by comparative qRT-PCR. Expression levels were normalized *versus* two housekeeping genes, HGPRT and 18 S. In (**c**) and (**e**), the reported values are the means of three independent experiments±S.D.; the value of proliferating myoblasts (P) is set equal to 1. *P-value <0.05; **P<0.01; ***P<0.001

The effect of p53 loss on metabolism and basal autophagy was investigated in myoblasts derived from p53 null mice. When p53 was genetically knocked out, the differentiation as well as the fusion index were inhibited^5, 6^ (data not shown). p53 deprivation had a clear impact on the balance between aerobic respiration and glycolysis during myogenesis. In the latest stages of differentiation, a significant increase of the levels of lactate (Figure 8a), as well as of the absolute concentration of ATP+ADP (Figure 8b) (48 and 72 h), was observed The complete spectrum of metabolites is shown in Supplementary Table S1B. In p53 null myoblasts, the mtDNA copy number (Figure 8c), as well as COXIV and oxphos protein levels (Figure 8d), do not increase during differentiation and mitochondria do not form tubular structures or networks (as in wild-type cells) but aggregates (Supplementary Figure S3). PGC-1*α*, significantly upregulated during differentiation in wild-type cells, fails to increase in p53 null cells (Figure 8e). Similar to wild-type cells, the PPP pathway is inhibited during differentiation as shown by decreased levels of G6PD mRNA (Supplementary Figure S4A) and reduced/oxidized glutathione ratios (Supplementary Figure S4B) as a function of the differentiation time.

As observed in wild-type myoblasts, in p53 null myoblasts the transcript levels of LC3 and FOXO3 increased during differentiation (Supplementary Figures S5A and B). It is unlikely that p53 is involved in their activation because, in this same time window, the level of p53 decreases^18^ and, when p53 is stabilized by nutlin, the mRNA levels of LC3 are unchanged, whereas the p53 transcriptional target Apaf-1 (that is, downregulated during myogenesis, Supplementary Figure S5C) is actively transcribed during differentiation.

**Figure 9:**
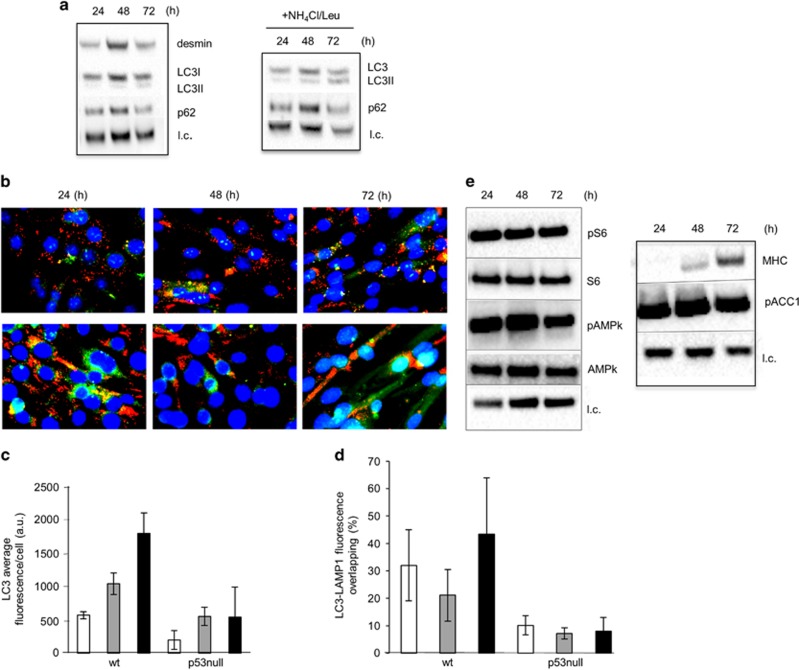
Autophagy is attenuated in the absence of p53 null cells and lysosomal activity is less efficient during myogenesis**. (a**) Left: WB analysis of desmin (53 kDa), LC3-I (18 kDa) and LC3-II (16 kDa) and p62 (62 kDa) performed on whole-cell extracts from p53 null cells during differentiation. Right: to measure the autophagic flux a mix of ammonium chloride and leupeptin (NH_4_Cl/Leu 20 mM/100 *μ*M) was added to the medium 2 h before cell harvest. (**b**) Representative fluorescence microscopy images of p53 null muscle cells during differentiation. (Top) LC3 (green) and Lamp1 (red); (Bottom) p62 (red) and Beclin 1 (green). The nuclei were stained with DAPI. ImageJ quantification of fluorescence intensity of LC3 (**c**) and of LC3-Lamp1 (yellow area) *versus* LC3 (green area) (**d**) in wt and p53 null muscle cells was performed as described in Materials and methods section. Statistical analysis was performed by one-way ANOVA followed by Newman–Keuls test. *P=0.05, **P<0.01, ***P<0.001. (**e**) WB analysis of pS6, S6 (32 kDa), pAMPk and AMPK (62 kDa) (left) and MHC (200 kDa) and p-ACC (280 kDa) (right) in whole-cell extracts of p53 null muscle cells during differentiation (24, 48 and 72 h in DM); l.c.=loading control

The raise in autophagy during myogenesis of p53 null myoblasts appears greatly attenuated, as indicated by the low rate of LC3-I/LC3-II conversion and of accumulation of LC3-II in the presence of lysosome inhibitors and the relative stability of p62 (Figure 9a). The muscle-specific marker, desmin, did not increase during differentiation supporting a role of p53 in myogenesis (Figure 9a). Consistent with a low basal autophagic flux, p53 null myotubes showed reduced expression of Beclin 1 in terms of fluorescence-positive aggregates (top panel) and increased accumulation of p62 (bottom panel) (Figure 9b). When the fluorescence associated with LC3 was quantified (Figure 9c), the kinetics of vacuolar LC3 levels (that is, autophagosome accumulation) showed similar levels during differentiation confirming an attenuated autophagic flux during differentiation of p53 null cells. Notably, p53 null cells (at any time of the differentiation process) presented with abnormalities in the acidic compartments (Figure 9b). The lysosomes appear enlarged and scattered throughout the cells. It is known that when autophagy is induced, lysosomes are recruited at the microtubule organizing center in order to fuse with autophagosome and form the autolysosomes.^19^ Consistently, the proportion of green area (LC3, autophagosomes) that overlapped with the yellow area (LC3-Lamp1, autophagolysosomes), assumed as an estimation of the capacity of autophagosomes to fuse with lysosomes, amounted to approximately 6% (in p53-wt MSCs this area was 5 fold bigger) (Figure 9d). Our data indicate that loss of p53 negatively impact on the autophagy–lysosomal system in MSCs. The mTORC1 pathway is active during differentiation also in p53 null myoblasts (Figure 9e). The levels of the Thr172 -pAMPk (left panel) and of its substrate pACC1 (right panel) did not increase in p53 null cells. Interestingly, high levels of pAMPk and of its substrate pACC1 were detected in p53 null cells already at 24-h differentiation time.

## Discussion

### The integration of metabolic and differentiation pathways

We show that our *in vitro* cell system faithfully mimics the metabolic reprogramming that is required for the divergent functions of myoblasts and myotubes. A reconfiguration of metabolic programs toward oxidative phosphorylation (OXPHOS) with increased mitochondrial biogenesis and activity is observed when myoblasts fuse into multinucleated myofibers, indicating that these processes are cell autonomous.

When p53 is genetically ablated, mitochondrial biogenesis is impaired and glycolysis prevails. p53 has been shown to influence metabolic pathways through several mechanisms,^20^ and the control of muscle metabolism reflects one of the functions of basal levels of p53.

An increase in the total level of glutathione and also of GSH was detected when myoblasts were shifted to DM for 24 h in agreement with previous studies.^21, 22^ The GSH content increase could represent a compensatory response to elevated ROS levels occurring during differentiation. A decrease of G6PD expression level and, consequently, of the glutathione redox balance is observed in wild-type and p53 muscle cells, when they reach terminal differentiation, indicating a tight control of the PPP in muscle.^12^

It is well established that p53 knockdown impairs myogenic differentiation via downregulation of pRb.^5, 6^ The impairment of mitochondrial function and activity that we observe in the absence of p53 may contribute to defective myogenesis as well. The abrogation of p53 manifests as impairment of PGC-1*α* transcriptional stimulation during *in vitro* differentiation and, interestingly, a reduction of the expression of PGC-1*α* in conjunction with diminished mitochondrial content and functionality was also reported in the gastrocnemius muscle extracted from p53 KO mice compared with its wild-type counterpart.^8^ The p53 null animals showed greater fatigability and less locomotory endurance than wild-type animals indicating that mitochondrial biogenesis and muscle performance are causally associated *in vivo* as well as *in vitro*. In agreement with a p53-mediated PGC-1 *α* regulation, it has been reported that p53 binds to the promoter of PGC-1*α* of both human and mouse genes and this event is positively related to increased PGC-1α expression and abrogated by inhibiting nitric oxide synthase.^23^ These data are somehow in contrast with data showing that p53 activated by telomere dysfunction binds and represses PGC-1*α* promoter.^24^ Thus, the interaction between p53 and PGC-1*α* appears to be specific to the cellular milieu.^25^ A new role for PGC-1*α* as a ‘guardian' of the normal mitophagic flux during myogenesis by blocking an excessive ROS production has been recently proposed.^26^ It is likely that the downregulation of PGC-1*α* when p53 is defective by increasing ROS production contributes to the alteration of the mitochondrial network as observed in p53 null myotubes.

### The integration of autophagy and metabolic pathways

Autophagy is intimately linked with cell metabolism^27^ and its homeostatic role in preserving muscle mass has been well documented.^28, 29^ This process is of particular importance in long-lived post-mitotic cells such as skeletal muscle fibers to adapt to different forms of cellular stresses by operating a lysosome-mediated degradation of obsolete and super-oxidized self-structures. The regulation of autophagy in muscle cells subjected to acute exercise^30, 31^ and upon nutrient starvation^32, 33^ have been clearly dissected, but little is known about the regulation of basal autophagy during myogenesis. Here we show that when myoblasts differentiate, autophagy is activated as a part of the metabolic reprogramming. Autophagy is upregulated soon after the myoblasts are cultured in DM, and thereafter keeps ongoing in myotubes. However, in myocytes that cannot fuse into myotubes, the autophagy flux is not hyper-modulated. In terminally differentiated myotubes, autophagosomes accumulate, despite their production is reduced, mainly because of inefficient fusion with lysosomes. This fact could be attributed to an insufficient pool of lysosomes available for autolysosome formation. Indeed, biogenesis and reformation of lysosomes in cells in which autophagy is upregulated require much time and energy.^34^

In contrast with most cell types, in skeletal muscle, autophagy was suggested to be independent of mTORC1 but controlled by FoxO3.^35, 36^ Yet, more recently, evidence has been provided that also in skeletal muscle mTORC1 is the dominant regulator of autophagy induction and ensures a tight coordination of metabolic pathways.^37^ Here we show that, when autophagy is activated during differentiation, S6 ribosomal protein is still phosphorylated. Thus, in contrast to starvation-induced autophagy, the mTORC1 kinase signaling pathway is active during myogenesis. Even more, the complete inhibition of mTORC1 by rapamycin and the associated increase of the autophagic flux in myotubes supports the view that mTORC1 has a tonic inhibition on autophagy under basal conditions. Our findings are in line with previously reported observations of mTOR inactivation-independent autophagy during the differentiation of C2C12 cells.^2^

AMPkα1 catalytic subunit potentiates myogenin expression and myogenesis.^38^ We observe that AMPkα is activated in the late stages of muscle differentiation as indicated by phosphorylation of AMPkα and its downstream target, ACC1, likely to meet the demands of ATP consumption.^39^ Activated AMPk has been shown to interact with mTOR thus suppressing mTORC1 activity and indirectly promoting autophagy,^40^ but it can also directly enhance autophagy by activating ULK1.^41^ As during myogenesis, mTORC1 and AMPk are both active, we favor the model where energy sensing, AMPk, is directly connected via ULK1 to basal autophagy during myogenesis.

The role of p53 on autophagy depends on its subcellular localization as well as on the cellular context.^11^ Here, we show that total abrogation of p53 in MSC-derived myoblasts leads to a lower autophagic flux as shown by accumulation of p62. A clear reduction in the number of lysosomes and autophagolysosomes was also observed in the absence of p53. To further support this observation, attenuated lysosomal capacity and lower autophagic flux was recently reported in resting muscles of p53 KO mice.^8^ Intriguingly, the genetic ablation of cathepsin D in zebrafish reflected in dystrophic development of the skeletal musculature.^42^ Here we show that p53 null myoblasts imperfectly develop into myotubes. We speculate that it is the clearance of damaged mitochondria (mitophagy) that is specifically affected in the absence of p53 as shown by accumulation of abnormal mitochondria in p53 null myotubes (Supplementary Figure 3S).^43^ The lysosomal–mitochondrial axis where oxidative stress produced by damaged mitochondria would lead to lysosomal rupture in a loop system^44^ may provide a mechanistic basis for attenuation of autophagy and thus imperfect myogenesis in the absence of p53. Interestingly, when p53 is lacking, baseline levels of AMPk activation are higher than in wild-type cells but there is no further increase during myogenesis. The augmented AMPk activation at baseline level may represent an adaptive response to the presence of mitochondrial damage and energetic stress.^45^ In response, AMPk would promote catabolic pathways to generate more ATP in agreement with the switch to glycolysis and increased ATP levels observed in p53 null cells. AMPk activation induces phosphorylation of p53 on serine 15 and this phosphorylation is required to initiate a metabolic AMPk-dependent cell cycle arrest^46^ that responds to glucose availability and occurs despite active mTOR.^47^ We speculate that, in the absence of p53, AMPk-dependent autophagy is not activated. In this scenario, AMPk would coordinates cell fate (i.e. proliferation, autophagy) with metabolic resources.

### The integration of autophagy and differentiation pathways

**Figure 10:**
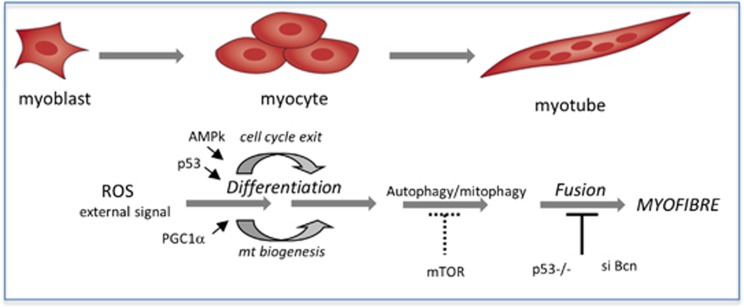
Scheme of the pathways operating during skeletal muscle differentiation. Active p53 is required when myoblasts live the cell cycle; concomitantly, AMPk *α*1 activation occurs and a switch from glycolysis to aerobic metabolism takes place. We speculate that a metabolic AMPk-dependent cell cycle exit and expression of muscle-specific proteins occur when mTOR is active and is coupled to stimulation of autophagy (likely mitophagy). In the constitutive absence of p53, differentiation-associated AMPk activation and mitochondrial biogenesis are impaired as well as autophagy. The ablation of p53 as well as Beclin 1 silencing affect the fusion step into multinucleated myotubes

Autophagy has a fundamental role in organ development and cell differentiation^5^ but no data are available on the functional role of basal autophagy during myogenic differentiation. We provide robust evidence that autophagy is an important part of successful muscle differentiation. As shown in Figure 10, skeletal myogenesis proceeds *in vitro* through a highly ordered sequence of events culminating in the cell fusion to form multinucleated myotubes. We found that myoblasts that have been silenced for Beclin 1 proceed through all these events with the exception of the fusion step that is significantly reduced. Similarly, inhibition of autophagy by NAC impacts on the fusion step. Under our NAC treatment conditions, the increased levels of ROS produced during differentiation are significantly inhibited. Whether the effect of NAC on autophagy is solely because of its ROS scavenging activity^16^ and/or to its role in GSH synthesis^48^ deserves further investigations. In support of an interdependence of autophagy and differentiation, myocytes, which are fully differentiated mononucleated cells that did not undergo fusion, were unable to rise the basal level of autophagy. We conclude that a fine tuning and balancing of autophagy is critical to allow the correct development of the skeletal muscle tissue, as it functions to maintain a close time-related connection between the synthesis of differentiation-associated proteins with the fusion of myocytes into myotubes. Successful differentiation of satellite-derived myoblasts into functional myotubes is a fundamental prerequisite for muscle regeneration, a repair process that is of primary importance in maintaining muscle function.^49, 50^ Our findings provide a better understanding of the gene networks operating during muscle differentiation thus opening new avenues for the therapies of muscle disorders and injuries.

## Materials and Methods

### Cell growth conditions and treatment

Mouse (strain FVB) MSC-derived myoblasts were a generous gift of M Crescenzi. Mouse (strain C3H) MSC-derived p53 null myoblasts were a kind gift of S Soddu. Both cell lines were isolated, cultured and differentiated as described in Tiainen *et al.*^51^

Lysosomal inhibitors (20 mM ammonium chloride plus 100 *μ*M leupeptin) were added 2 h before cell collection. NAC (5 mM f.c.) and rapamycin (20 nM f.c.) were re-added every 24 h. All reagents were purchased from Sigma (St Louis, MO, USA).

The differentiation index was calculated as % nuclei in myosin heavy chain (MHC)-positive cells/total number of nuclei and the fusion index as the average number of nuclei in MHC-positive cells/total number of MHC-positive cells.

### Mitochondrial DNA copy number and gene expression analysis

Relative mitochondrial DNA copy number was analyzed by absolute QPCR using TaqMan probes. Mitochondrial and nuclear DNA were detected by using ND2 and Rplp0 single tube Taqman real-time PCR assay, respectively (cat. no. 4331182; Life Technologies, Austin, TX, USA).

RNA was extracted by RNeasy kit (cat. no. 74106; Qiagen, Hilden, Germany) and cDNA synthesis was carried out by the high capacity cDNA reverse transcription kit (cat. no. 4368813; Life Technologies). Gene expression analysis was carried out using single tube Taqman real-time PCR assays (cat. no. 4331182; Life Technologies).

### RNA interference

Beclin 1 siRNA oligonucleotides (Dharmacon, Chicago, IL, USA) were used for silencing (20 nM f.c.). Transfection was performed using Interferin (Polyplus Transfection, Inc., New York, NY, USA). The efficacy of silencing was monitored by QPCR and WB. Myoblasts were seeded in 100 mm or 35 mm gelatin-coated plates in growth medium (GM) and silenced the day after seeding and shifted in DM immediately after transfection.

### IF analysis

Cells were seeded in slide flasks gelatin coated and fixed for 10 min by cold absolute methanol at the indicated times. Primary and secondary antibodies are described in Supplementary Information. Glass cover slides were mounted with a drop of ProLong Gold antifade reagent containing DAPI (Molecular Probes Invitrogen, Monza, Milano, Italy). Mitochondrial morphology was analyzed by staining with 40 nM Mito Tracker green (Thermo Fisher Scientific Inc., Monza, Milano, Italy, M7514).

Images were taken on an inverted microscope equipped with a confocal spectral imaging system (Olympus Fluoview 1000, Olympus, Tokyo, Japan) and, for some experiments, with a fluorescence microscope (Leica, Wetzlar, Germany, DMI6000B). Emitted fluorescence was recorded during single excitation sessions in the same field and same conditions for stack images collection. The ImageJ software (http://rsbweb.nih.gov/ij/) was used for quantification of the fluorescent signal.^47^

### Protein analysis

Cell homogenates were prepared by ultrasonication in a buffer containing detergents and proteases and phosphatases inhibitors (Roche Applied Science, Mannheim, Germany). Proteins were separated on NuPAGE 4–12% and 3–8% NuPAGE Tris-Acetate precast polyacrylamide gels (Life Technologies) and analyzed by WB using the antibodies described in Supplementary Information. Immunocomplexes were revealed by using a peroxidase-conjugated secondary antibody (Bio-Rad, Richmond, CA, USA), as appropriate. WB were developed by using the West Dura kit (Pierce Chemical, Rockford, IL, USA). The results were visualised and estimated by Chemi Doc XRS+ with Image lab Software (Bio-Rad). A loading control HSP90 was used.

### NMR spectroscopy

Cells were washed twice with ice-cold physiological saline solution and pellets suspended in 0.5 ml of ice-cold twice-distilled water. Aqueous extracts were processed as previously described.^52^ High-resolution NMR experiments (25 °C) were performed at 9.4 T and 16.4 T (Bruker AVANCE spectrometers, Bruker GmbH, Karlsruhe, Germany). 1H-NMR spectra of cell extracts were acquired using 90° flip angle, 30-s repetition time, 32 K time domain data points and 128 transients.

The amount of intracellular ATP was also measured by a luciferin/luciferase-based assay (Roche Applied Science).

### GSH and GSSG measurement

Sample aliquots (1x10^6^ cells/ml) were deproteinized by adding 1.22 M iced trichloroacetic acid (1:2  v/v), kept 5 min in ice and centrifuged at 10 000 r.p.m. for 5 min at 4 °C. GSH was determined spectrophotometrically at 412 nm, in the clear supernatants 5 min after the addition of 0.1 mM 5,5′-dithiobis-(2-nitrobenzoic) acid (DTNB) reagent to 0.1 mM phosphate buffer/1 mM diethylenetriaminepentaacetic acid, pH 7.4, containing aliquots of samples or GSH standard curve. GSSG was measured in 25 *μ*l cleared acidified supernatants by using DTNB-GSSG reductase recycling assay.^53^ GSH and GSSG content were normalized for cell number.

### EPR measurement of ROS levels

Cells were washed twice with phosphate-buffered solution (PBS), pH 7.4, and pellets suspended to obtain a concentration of 10 × 10^6^ cells/ml. The spin probe 1-hydroxy-3-carboxy-2,2,5,5-tetramethylpyrrolidine (CPH; ENZO Life Sciences Inc., Lausen, Switzerland) was dissolved in degassed PBS without Ca^2+^ and Mg^2+^ (Sigma D5652), pH 7.4, extensively treated with Chelex-100 (Bio-Rad) to avoid metal contamination. In all, 0.5 mM CPH was added to 100 *μ*l myoblast suspensions. The oxidation of CPH was monitored by the formation of the characteristic three-line spectrum with hyperfine coupling constant of 1.63±0.04 mT attributable to the corresponding nitroxide radical 3-carboxyproxyl radical (CP•). Samples were drawn up into a gas-permeable teflon tube with 0.81 mm internal diameter and 0.05 mm wall thickness (Zeuss Industrial Products, Raritan, NJ, USA). The teflon tube was inserted into a quartz tube and fixed to the EPR cavity. Spectra were acquired 20 min after the addition of the spin probe at 37 °C. The low field shoulder of this spectrum was chosen to quantify the CP• because the middle component centered at g 2.0 overlaps with other free radical signals found in biological systems. Spectra were acquired with a Bruker E-scan continuous wave X-band benchtop spectrometer (Bruker, Rheinstetten, Germany) with the following instrumental settings: modulation frequency, 100 kHz; microwave frequency, 9.7 GHz; microwave power, 10 mW; modulation amplitude, 0.1 mT; conversion time, 20.5 ms; time constant, 82 ms; sweep time, 21 s; number of scans, 60.

## Abbreviations

ACC1: acetyl CoA carboxylase
AMPk: 5' adenosine monophosphate-activated protein kinase
Atg 5: autophagy protein 5
Becn1: beclin 1
COXIV: cytochrome c oxidase
DM: differentiation medium
EPR: electron paramagnetic resonance
ER: endoplasmic reticulum
FOXO3: forkhead box O3
G6PD: glucose-6-phosphate dehydrogenase
GM: growth medium
GSH: reduced glutathione
GSSG: oxidized glutathione
IF: immunofluorescence
Lamp1: lysosome-associated membrane protein
LC3: light chain 3
MHC: myosin heavy chain
MSC: muscle satellite cell
mTOR: mammalian target of rapamycin
mTORC1: mammalian target of rapamycin complex 1
NAC: *N*-acetyl cysteine
NMR: nuclear magnetic resonance
oxphos: succinate ubiquinone oxydoreductase
PGC-1*α*: peroxisome proliferator-activated receptor gamma coactivator 1 alpha
PGC-1*β*: peroxisome proliferator-activated receptor gamma coactivator 1 beta
PPP: pentose phosphate pathway
S6: ribosomal S6 subunit
WB: western blot
