# Supplementary figures and images for: The fine tuning of metabolism, autophagy and differentiation during in vitro myogenesis
Source: Cell Death Dis. 2016 Mar 31;7(3):e2168–. doi: 10.1038/cddis.2016.50 (PMC4823951; doi:10.1038/cddis.2016.50)

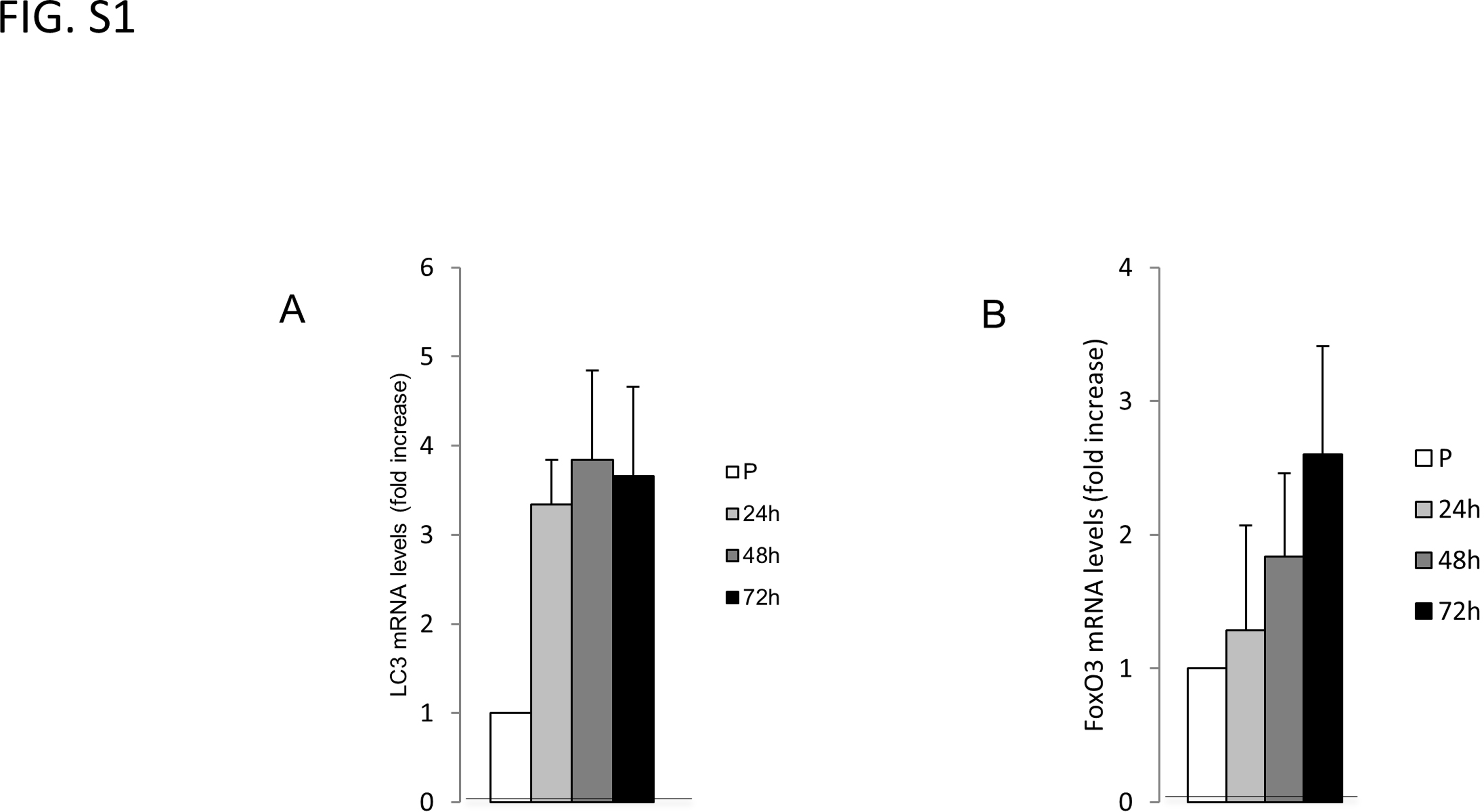

Supplement: Supplementary Figure S1 [file cddis201650x1.tif]

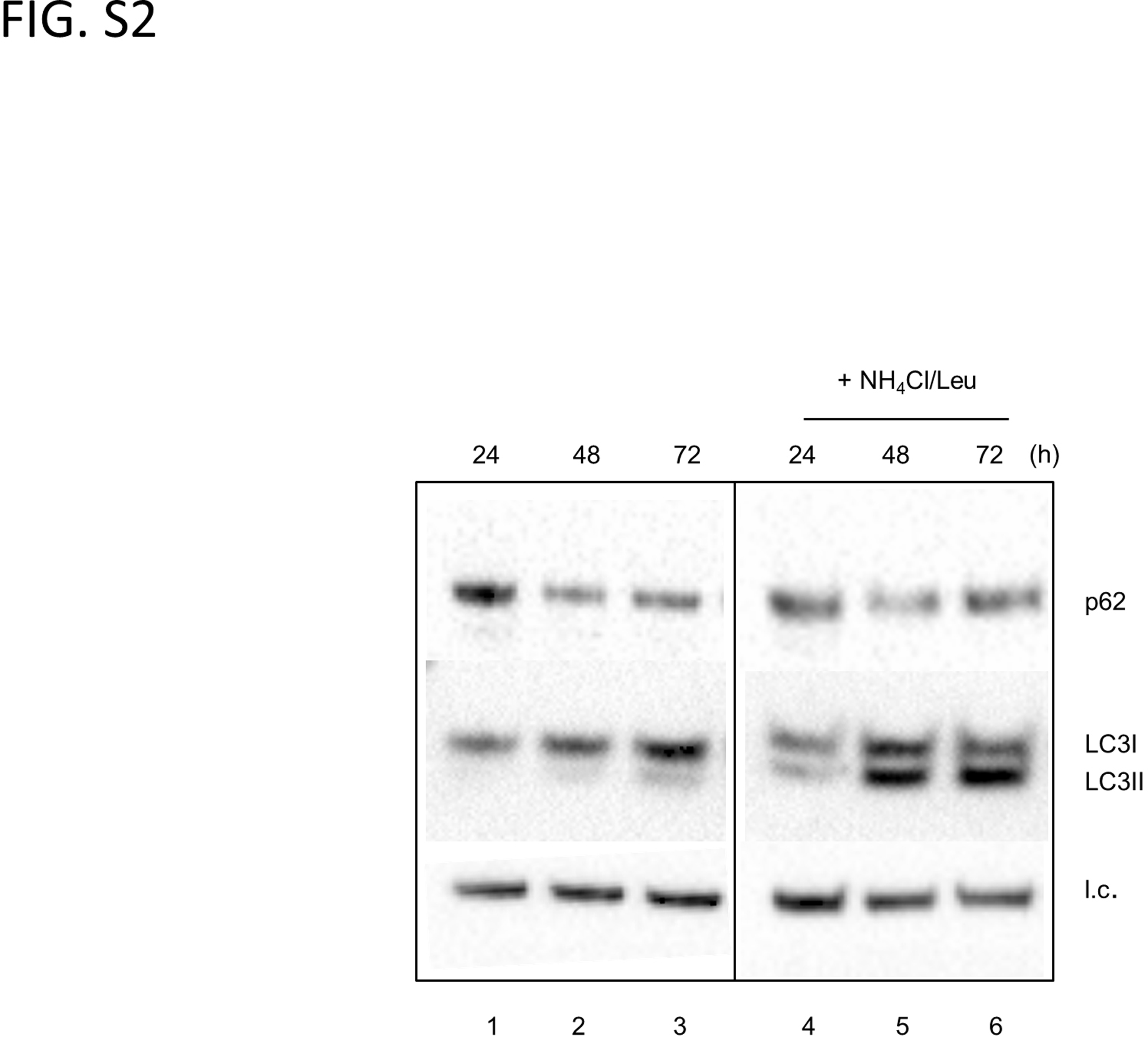

Supplement: Supplementary Figure S2 [file cddis201650x2.tif]

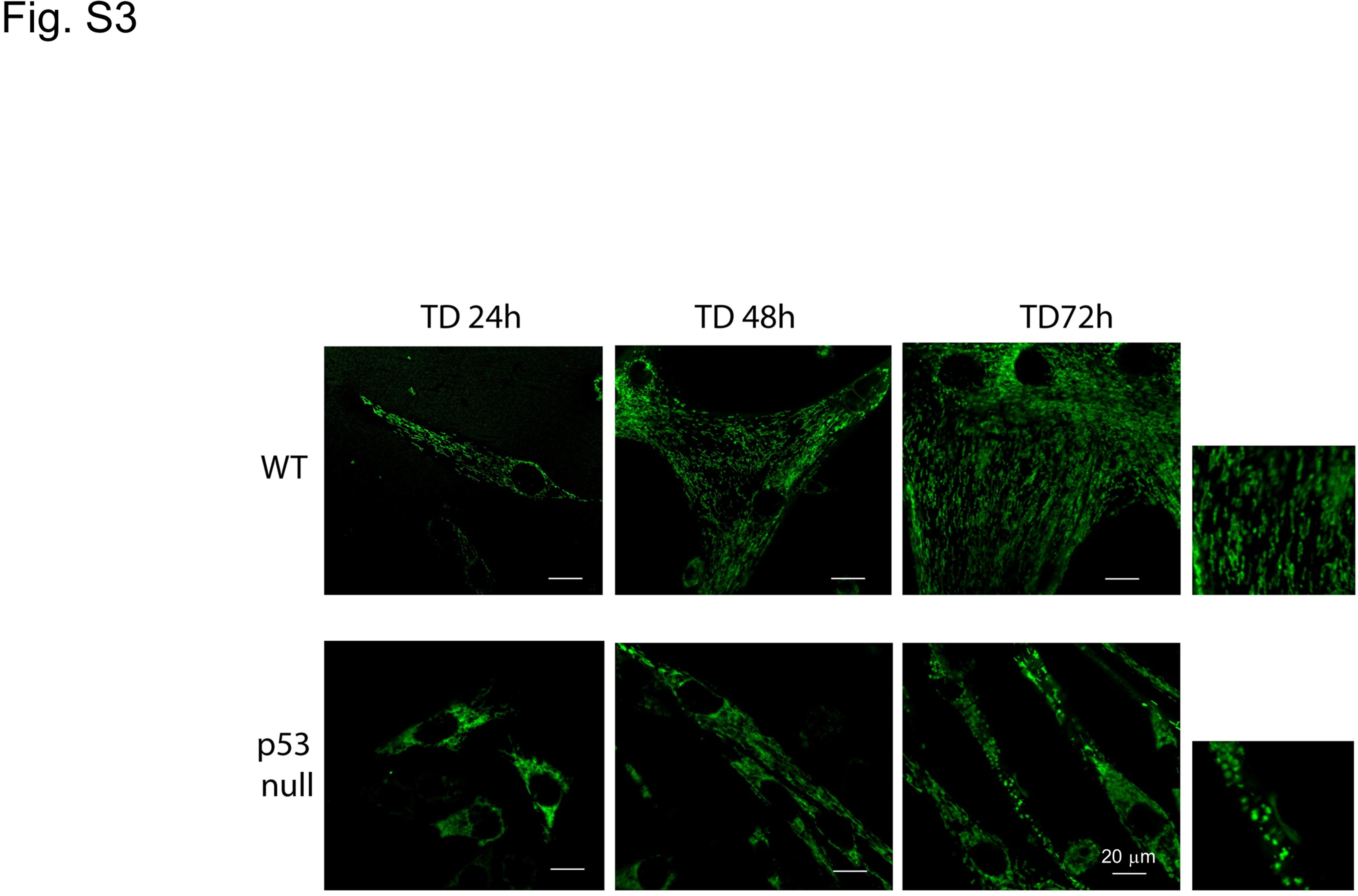

Supplement: Supplementary Figure S3 [file cddis201650x3.tif]

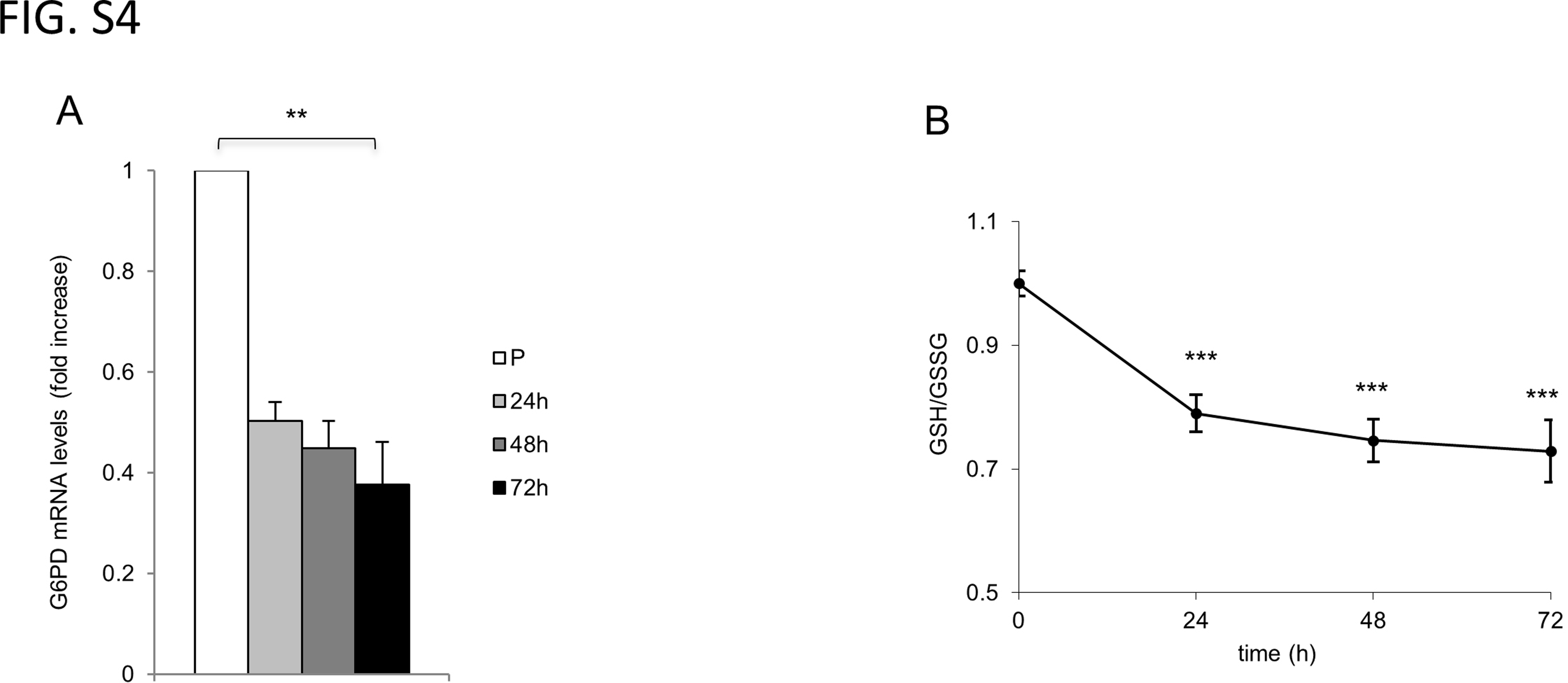

Supplement: Supplementary Figure S4 [file cddis201650x4.tif]

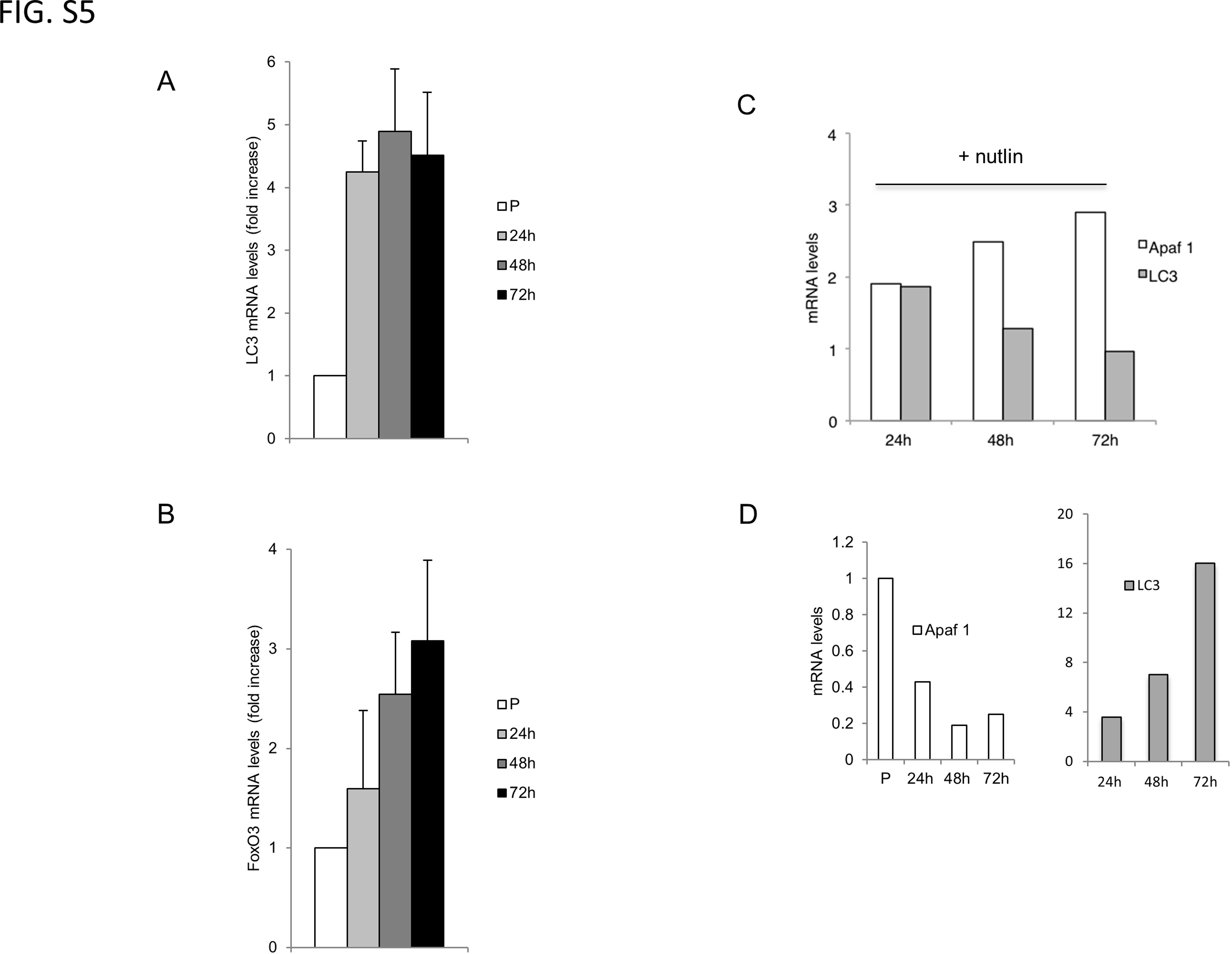

Supplement: Supplementary Figure S5 [file cddis201650x5.tif]
