# Supplementary material for: The fine tuning of metabolism, autophagy and differentiation during in vitro myogenesis
Source: Cell Death Dis. 2016 Mar 31;7(3):e2168–. doi: 10.1038/cddis.2016.50 (PMC4823951; doi:10.1038/cddis.2016.50)
Supplement: Supplementary Information [file cddis201650x6.doc]

**SUPPLEMENTAL INFORMATION**

**Figure S1**

**Gene reprogramming during muscle differentiation involves autophagy-related genes.** Analysis of mRNA levels of the autophagy genes LC3 (A) and FoxO3 (B) in muscle cells during differentiation (after 24, 48 and 72h growth in DM) as determined by qRT-PCR. Expression levels were normalized *versus* two housekeeping genes, HGPRT and 18S. Relative expressions are shown with respect to the basal level of proliferating myoblasts (P) which was set equal to 1. The reported values are the means of three independent experiments ± SD.

**Fig. S2**

**The induction of autophagy during skeletal muscle differentation is confirmed in primary myogenic cell cultures.** Western blot analysis of the autophagy markers LC3-I (18 kDa), LC3-II (16 kDa) and p62 (62 kDa) in wild-type primary myogenic cells grown in DM at the indicated times (lanes 1-3). The autophagic flux inhibitors, ammonium chloride and leupeptin (NH_4_Cl /Leu, 20mM/100microM), were added 2h before cell harvest (lanes 4-6); l.c.=loading control.

**Figure S3**

**Mitochondria of p53 null myotubes are structurally abnormal***. In vivo* imaging of the mitochondrial network by confocal microscopy. Wild-type (wt) and p53 null terminally differentiated (TD) muscle cells were incubated with MitoTracker green probe for 30 min before image collection. In normal myotubes mitochondria form tubular structures or networks while mitochondrial aggregates are visible in p53 null myotubes

**Figure S4**

**G6PD expression levels and glutathione redox balance in p53 null muscle cells during differentiation.** (A) Comparative analysis during differentiation of mRNA levels of glucose-6-phosphate dehydrogenase (G6PD) as determined by qRT-PCR. Expression levels were normalized versus two housekeeping genes, HGPRT and 18S. Relative expressions are shown with respect to the basal level of the proliferating myoblasts which is set equal to 1. The reported values are the means of three independent experiments ± SD. (B) GSH and GSSG ratio obtained by enzymatic assay titration (mean ±SD for n≥3). Statistical analysis was performed to compare the values obtained in proliferating myoblasts (P) with those obtained in differentiated cells (24-72 h in DM). *p value <0.05; **p<0.01; ***p<0.001.

**Figure S5**

**Gene reprogramming of autophagy-related genes occurs also during differentiation of p53 null myoblasts.** Analysis of mRNA levels of the autophagy genes LC3 (A) and FoxO3 (B) in p53 null cells during differentiation (after 24, 48 and 72h growth in DM) as determined by qRT-PCR. Expression levels were normalized *versus* two housekeeping genes, HGPRT and 18S. Relative expressions are shown with respect to the basal level of proliferating myoblasts (P) which was set equal to 1. The reported values are the means of three independent experiments ± SD. (C) Comparative gene expression levels of Apaf1 and LC3 during differentiation time in the presence of nutlin-3. The p53 stabilizer was freshly renewed every 24h. The expression levels of the samples differentiated in the presence of nutlin were set equal to 1 at each time point. (D) Gene expression levels of Apaf1 and LC3 during differentiation time in the presence of nutlin-3.

Table S1: NMR quantification of intracellular metabolites involved in glucose, phospholipid and energetic metabolism in wild type (wt), and p53 null myoblasts (time 0) and during differentiation (24h, 48h, 72h).

| Metabolite^a^ | wt/0h | | p53 null/0h | |  | wt/24h | | p53 null/24h | |  |
| --- | --- | --- | --- | --- | --- | --- | --- | --- | --- | --- |
|  | MEAN^b^ | SD | MEAN^b^ | SD | p | MEAN^b^ | SD | MEAN^b^ | SD | p |
| mio-ins | 11.70 | 5.03 | 30.66 | 21.20 | 0.14 | 20.16 | 1.25 | 45.24 | 17.08 | 0.06 |
| Glycine | 4.95 | 1.37 | 5.93 |  |  | 28.04 | 19.65 | 18.53 | 1.66 | 0.56 |
| Tau | 21.72 | 9.38 | 18.56 | 3.88 | 0.61 | 47.13 | 6.94 | 49.51 | 17.35 | 0.79 |
| tCr | 11.73 | 4.25 | 5.29 | 0.25 | 0.04 | 19.43 | 9.10 | 15.57 | 6.34 | 0.50 |
| Asp | 9.89 | 7.92 | 16.44 | 3.35 | 0.39 | 19.01 | 12.04 | 28.64 | 20.19 | 0.60 |
| GS | 16.87 | 8.86 | 28.51 | 4.92 | 0.12 | 31.14 | 4.62 | 56.37 | 24.13 | 0.14 |
| Gln | 21.08 |  | 24.85 |  |  | 11.97 |  | 41.91 |  |  |
| Glt | 16.96 | 6.91 | 45.31 | 16.70 | 0.01 | 28.75 | 6.34 | 58.32 | 38.06 | 0.18 |
| Succ | 2.14 | 2.07 | 1.62 |  |  | 2.82 | 2.01 | 3.63 |  |  |
| Glx | 49.42 | 25.67 | 90.39 | 19.63 | 0.06 | 86.27 | 16.66 | 143.70 | 75.08 | 0.19 |
| Acetate | 4.48 | 1.81 | 7.53 | 3.38 | 0.14 | 7.16 | 3.78 | 9.57 | 7.03 | 0.53 |
| Alanine | 9.89 | 4.47 | 12.90 | 3.11 | 0.35 | 18.39 | 6.33 | 34.71 | 18.07 | 0.10 |
| Lactate | 15.55 | 2.35 | 18.58 | 4.10 | 0.22 | 20.15 | 3.37 | 26.69 | 9.44 | 0.19 |
| Isoleucine | 3.14 | 2.21 | 4.28 | 2.35 | 0.67 | 4.35 |  | 4.20 | 3.76 |  |
| Valine | 4.76 | 4.18 | 6.41 | 1.52 | 0.65 | 4.57 |  | 5.38 | 3.24 |  |
| GPC | 0.75 | 0.18 | 0.70 | 1.06 | 0.95 | 2.19 | 1.48 | 0.88 | 0.81 | 0.19 |
| PCho | 8.66 | 3.03 | 5.85 | 2.88 | 0.24 | 11.49 | 4.87 | 17.66 | 7.24 | 0.17 |
| Cho | 0.51 | 0.40 | 0.38 | 0.50 | 0.71 | 1.26 | 1.04 | 1.24 | 1.03 | 0.98 |

| Metabolite^a^ | wt/48h | | p53 null/48h | |  | wt/72h | | p53 null/72h | | |  |
| --- | --- | --- | --- | --- | --- | --- | --- | --- | --- | --- | --- |
|  | MEAN^b^ | SD | MEAN^b^ | SD | p | MEAN^b^ | SD | MEAN^b^ | SD | | P |
| mio-ins | 24.31 | 1.37 | 64.18 | 8.42 | 0.00 | 23.26 | 8.06 | 54.95 | 15.67 | | 0.02 |
| Glycine | 21.96 | 19.32 | 49.82 |  |  | 27.67 | 0.67 | 39.69 |  | | 0.14 |
| Tau | 42.35 | 3.31 | 67.72 | 4.31 | 0.00 | 37.66 | 14.33 | 52.82 | 8.17 | | 0.09 |
| tCr | 37.87 | 8.23 | 35.58 | 3.79 | 0.63 | 39.34 | 20.39 | 39.27 | 7.92 | | 1.00 |
| Asp | 17.87 | 1.31 | 32.61 | 5.03 | 0.01 | 19.23 | 6.92 | 32.15 | 10.10 | | 0.13 |
| GS | 36.21 | 0.64 | 80.29 | 10.14 | 0.00 | 31.31 | 13.60 | 60.60 | 19.15 | | 0.05 |
| Gln |  |  | 48.62 |  |  | 28.38 |  | 60.03 |  | |  |
| Glt | 39.35 | 11.91 | 118.75 | 16.59 | 0.00 | 38.00 | 17.83 | 109.89 | 29.97 | | 0.00 |
| Succ | 4.22 |  | 3.57 |  |  | 3.92 | 1.23 | 5.28 |  | |  |
| Glx | 101.08 | 17.35 | 250.22 | 37.63 | 0.00 | 99.40 | 32.88 | 231.67 | 65.39 | | 0.01 |
| Acetate | 8.49 | 2.68 | 11.20 | 5.09 | 0.34 | 7.33 | 4.19 | 8.22 | 1.71 | | 0.70 |
| Alanine | 21.84 | 4.76 | 64.56 | 2.40 | 0.00 | 26.20 | 12.25 | 52.41 | 15.94 | | 0.02 |
| Lactate | 19.87 | 4.80 | 43.70 | 9.64 | 0.00 | 23.42 | 9.63 | 36.59 | 12.35 | | 0.09 |
| Isoleucine | 5.31 |  | 9.48 | 3.91 |  | 4.09 | 1.23 | 11.61 | 1.69 | 0.04 | |
| Valine | 6.42 |  | 9.66 | 2.36 |  | 4.49 | 0.31 | 11.90 | 0.43 | 0.00 | |
| GPC | 3.95 | 4.92 | 2.39 | 1.46 | 0.57 | 5.13 | 2.24 | 3.14 | 0.24 | 0.29 | |
| PCho | 14.30 | 6.68 | 23.33 | 13.56 | 0.28 | 6.47 | 3.76 | 16.25 | 7.30 | 0.02 | |
| Cho | 1.79 | 1.36 | 1.22 | 1.10 | 0.56 | 1.29 | 1.01 | 1.28 | 1.32 | 0.99 | |

^a^Abbreviations: asp, aspartate Cho, free choline; Glx, glutamate(glt) plus glutamine (gln) and glutathione (GS); GPC, glycerophosphocholine; Lac, lactate; m-Ins, myo-inositol; PCho, phosphocholine; tau, taurine; tCr, total creatine (creatine plus phosphocreatine).

^b^The concentration of metabolites is reported as nmol/10^6^ cells, (mean± maximum deviation for n=2; ±SD for n≥3).

**Supplemental Experimental Procedures**

Isolation of murine skeletal muscle satellite cells (MSC) was conducted as described below. Briefly, skeletal MSC were isolated from the hind limb muscles of young FVB;129 mice. MSC were maintained as actively proliferating adult stem cells (myoblasts) and induced to differentiate in post-mitotic myotubes by modifying the cell growth conditions. In particular, myoblasts were sub-cultured in the growth medium (GM: F-10 nutrient mixture plus glutamax supplemented with 10% of fetal calf serum, 100 U/ml penicillin/streptomycin, 3% chicken embryo extract, obtained from 10- to 11-day-old embryos, and 2.5 mg/ml of recombinant human FGF-basic (Peprotech, Rocky Hill, NJ, USA). To induce terminal differentiation, the GM was replaced with the differentiation medium (DM:Dulbecco’s modified Eagle medium plus glutamax, supplemented with 10% foetal calf serum and 100 U/ml penicillin/streptomycin). Cells were incubated at 38°C, 10% CO_2_. The efficiency of cell differentiation was routinely controlled by immunofluorescence analysis with myosin heavy chain (MHC) antibodies and evaluation of the differentiation and fusion indexes. The differentiation index is calculated as the percentage of nuclei belonging to MHC-positive cells divided by the total nuclei. The fusion index is calculated as the average number of nuclei in MHC-positive cells (which contain three or more nuclei) divided by the total number MHC-positive cells.

Relative mitochondrial DNA copy number was analysed by absolute QPCR using TaqMan probes for mitochondrial ND2 and nuclear 36B4 genes. The mitochondrial genome copy number of the myoblasts was set as 1. Total DNA was isolated using QIAamp DNA mini kit (QIAGEN).

RNA extraction was carried out using RNeasy mini kit (QIAGEN). cDNA synthesis and relative gene expression analysis were carried out using the High Capacity cDNA reverse transcription kit and TaqMan real-time PCR assays purchased by Applied Biosystems accordingly to the manufacturer’s instructions.

*Immunofluorescence analysis*

Mitochondrial morphology was analysed by staining with 40 nM Mito Tracker green (Thermo Fisher Scientific Inc. M7514) for 30 min in saline solution.

Images were taken on an inverted microscope (Olympus) equipped with a confocal spectral imaging system (Olympus Fluoview 1000) using a (Olympus) planapo objective 60X oil A.N. 1,42. Excitation light was obtained by an Argon Ion Laser 488 nm or 594 nm for green or red fluorescence, respectively. Emitted fluorescence was recorded during single excitation sessions in the same field and same conditions for stack images collection.

*Protein expression analysis*

List of antibodies:

Mouse monoclonal anti-complex II 70 KDa Fp subunit antibody (Invitrogen 459200); rabbit polyclonal Cox IV antibody (Cell Signaling Technology, Inc. MA, USA #4844); rabbit monoclonal anti-S6 ribosomal protein (Cell Signaling Technology, Inc. MA, USA #2217), rabbit monoclonal anti phospho-S6 ribosomal protein (Cell Signaling Technology, Inc. MA, USA #4856), rabbit polyclonal anti-Beclin 1 (Santa Cruz Biotechnology, Inc. CA USA sc-11427), rabbit polyclonal anti-LC3 (MBL Medical & Biological Laboratories, Ltd., Japan), guinea pig polyclonal anti-p62 (Progen, Biotechnik GmbH,GP62-C, Germany), mouse monoclonal anti-desmin (Abcam, RD30, UK), polyclonal anti-myosin heavy chain (a kind gift from Dr. Marco Crescenzi), rabbit polyclonal anti-phospho-Acetyl-CoA carboxylase (Ser79) (Cell Signaling Technology, Inc. MA, USA #3661), anti-Acetyl-CoA carboxylase (Cell Signaling Technology, Inc. MA, USA #3662) rabbit polyclonal anti-AMPk alpha (Cell Signaling Technology, Inc. MA, USA # 2532), rabbit polyclonal anti-phospho-AMPk alpha (Thr172) (Cell Signaling Technology, Inc. MA, USA # 2535), HSP 90alpha/beta (Santa Cruz Biotechnology, Inc. CA USA sc-13119). HSP 90alpha/beta has been used as loading control (l.c.). Immunocomplexes were revealed by using a peroxidase-conjugated secondary antibody (Biorad), as appropriate, and subsequent peroxidase-induced chemiluminescence reaction (PerkinElmer,Waltham, MA USA).

NMR spectroscopy.

Aqueous extracts were prepared in EtOH 70% according to an established protocol (Pascucci et al., 2012). Briefly, samples were ultra-sonicated at 20 kHz by a MSE ultrasonic disintegrator Mk2 (Crawley, Sussex, UK) and centrifuged at 14000 x g for 30 min. Supernatants were lyophilized twice in a RVT 4104 Savant lyophilizer (Mildford), and the residue resuspended in 0.7 ml D2O (Sigma-Aldrich, St. Louis, MO) containing 0.1 mM 3-(trimethylsilyl)-propionic-2,2,3,3-d4 acid sodium salt (TSP) as internal standard. High-resolution NMR experiments (25°C) were performed at 9.4T and 16.4 T (Bruker AVANCE spectrometer, Bruker GmbH, Karlsruhe, Germany). ^1^H-NMR spectra of cell extracts were acquired using 90° flip angle, 30 s repetition time, 32K time domain data points and 128 transients.
